# Optimizing SloMo, a Digitally Supported Therapy Targeting Paranoia, for Implementation: Inclusive, Human-Centered Design Study

**DOI:** 10.2196/75377

**Published:** 2025-12-22

**Authors:** Thomas Gant, Kathryn M Taylor, Thomas Ward, Philippa Garety, Amy Hardy

**Affiliations:** 1 Department of Psychology Institute of Psychiatry, Psychology and Neuroscience King's College London London United Kingdom; 2 South London and Maudsley NHS Foundation Trust London United Kingdom

**Keywords:** co-design, design thinking, digital health, user-centered design, participatory design, mHealth, eHealth, schizophrenia, psychosis, user experience, engagement, therapy

## Abstract

**Background:**

Despite the promise of digital therapeutics in providing scalable interventions for psychosis, translating them from clinical trials to routine care is challenging. SloMo is an evidence-based, digitally supported cognitive behavioral therapy for psychosis comprising a web-based therapy platform and mobile app. The therapy encourages individuals to slow down for a moment, to address fast-thinking habits fueling paranoia. SloMo has received a NICE Early Value Assessment recommendation for use in the National Health Service to address evidence gaps related to its use in the real world, and an implementation study is underway.

**Objective:**

This study aimed to optimize the SloMo software for implementation by addressing limitations of the first release, reducing technology complexity, and improving user experience, to increase equitable outcomes.

**Methods:**

An inclusive, human-centered design methodology was used to optimize SloMo. The redesign sought to reduce the technology’s complexity and improve the user experience for diverse patients and therapists. The Double Diamond framework structured the iterative redesign, integrating insights from patient and public involvement consultants, therapists, and a transdisciplinary co-design team. The Double Diamond process was facilitated through 24 transdisciplinary workshops. These were supported by the following methods: identifying implementation barriers through desk research of SloMo’s evidence and qualitative interviews with experts by experience (n=2); redefining user needs; iteratively developing solutions through user testing sessions with service user consultants (n=32); and validating the minimum viable product through think-aloud testing sessions with therapist (n=10) and service user (n=11) consultants.

**Results:**

Users wanted a form of cognitive behavioral therapy for psychosis that was usable, trustworthy, enjoyable, personalized, normalizing, and memorable. The redesign, therefore, included a minimalist user interface, more diverse lived experience vignette content, enhanced agency over data, greater representation of protected characteristics and their intersectionality, and intuitive navigation. Feedback from a purposively sampled patient and therapist sample validated the redesign as it was associated with a strong user experience, particularly in relation to usability and usefulness.

**Conclusions:**

The study produced a redesign of the SloMo software optimized for real-world use, whilst retaining fidelity to the therapeutic content of the previous version. Through an inclusive, human-centered approach, the optimized design of SloMo addresses barriers to adoption by reducing complexity and fostering accessibility. This study underscores the value of integrating lived experience involvement into digital therapeutics to support scalable, equitable, and sustainable mental health care solutions.

## Introduction

Digital therapeutics present an opportunity for providing scalable, usable, and targeted evidence-based interventions for people with psychosis. Emerging evidence suggests digital technologies are acceptable and effective at addressing a range of treatment targets in this population [1,2]. With device ownership and levels of technology confidence on the increase, investment and interest in digital therapeutics for psychosis are growing. However, despite their promise, implementing digital therapeutics within real-world clinical settings following clinical trials is difficult, and a notable evidence-practice gap exists [3].

Successful implementation of digital technology in psychosis is multifactorial, with technology complexity being a particularly important barrier [4]. People from minoritized backgrounds are more likely to experience additional barriers to technology access and digital literacy, and if this is not given careful consideration, there is the risk that technology might inadvertently magnify health inequalities [5]. The National Health Service (NHS) Inclusive Digital Healthcare Framework [6] lists accessibility and ease of technology use as a key domain of digital inclusion. Inclusive, human-centered design (iHCD) is a method that seeks to ensure the design of a product aligns with the needs and capabilities of the people for whom it is intended [7]. iHCD uses ethnography to understand users and their contexts, with purposive recruitment to ensure representation across relevant user identities and characteristics (ie, gender, age, ethnicity, cognitive abilities, use of technology, and attitudes to therapy) and the inclusion of seldom-heard groups [8,9].

SloMo is a digitally supported psychological therapy targeting paranoia in psychosis. It was developed to address the limitations of conventional psychological therapies for psychosis; namely, issues in access, experience, and outcomes [10-12]. SloMo works by targeting fast thinking habits that fuel worries and supports people to slow down for a moment to find ways of feeling safer and living well. Specifically, SloMo adopts a causal-interventionist approach [13] to enhance belief flexibility (“slow thinking”) and build awareness of reasoning biases (“fast thinking”) that are characteristic of paranoia [14,15].

The technology comprises a web-based therapy platform that augments face-to-face individual therapy sessions. The platform offers interactive features such as animated psychoeducation videos, which encourage reflection, games promoting belief flexibility, and personalized content, including a psychological formulation, session summaries, and between-session task plans. Key content is synchronized with a mobile app that supports self-management in daily life. The mobile app supports users to notice worries and connect to alternative, safer thoughts through interactive features to promote slowing down, reminders of session content, and a record of between-session tasks. SloMo uses technology to support the visualization of thoughts and thinking habits. People can interact with their personalized SloMo thought bubbles, altering speed and size to reflect thinking habits and distress. SloMo tips support people to slow down and notice new information, helping to shrink fast-spinning gray, worry bubbles and grow colorful, slow-spinning helpful thoughts to promote well-being [16].

iHCD was used for the design of the first release of SloMo [16,17]. Ethnographic research insights indicated users with paranoia needed a form of cognitive behavioral therapy for psychosis (CBTp) that was usable, trustworthy, enjoyable, personalized, normalizing, and memorable, and the design solution was iteratively developed with them. The therapy design and usability of SloMo was validated by a comprehensive mixed methods approach [15,18-20]. Notably, although baseline technology use and confidence were lower in groups known to be disproportionately affected by the digital divide (Black people and older adults [21]), these baseline characteristics were not associated with differences in outcomes or user experience. This suggests the SloMo therapy design may bridge the “digital divide” and support accessibility, as intended.

In a multi-site randomized controlled trial (RCT) (N=362), SloMo demonstrated improved paranoia, self-concept, and well-being outcomes over 6 months compared with treatment as usual, with small to moderate effects [18]. SloMo received a NICE Early Value Assessment recommendation [22] for use in the NHS whilst real world data is collected to address identified evidence gaps, and accordingly is now being tested in a type II hybrid implementation-effectiveness study (ClinicalTrials.gov identifier: NCT06568081). McGinty et al [23] recommend that the evidence-practice gap be approached with a complexity science lens, including adaptation of the intervention to the implementation context. Consistent with this, we sought to optimize the SloMo therapy software for routine care prior to commencing our implementation-effectiveness study. The Non-adoption, Abandonment, Scale-up, Spread, Sustainability (NASSS) framework, an evidence-based implementation science framework for technologies, informed the work [24,25]. This mixed methods approach encourages the minimization of complexity in key domains (target problem, technology, adopters, organization, and broader system) to reduce the likelihood of nonadoption and abandonment, and increase the likelihood of scale-up, spread, and sustainability. The NASSS model has been used to plan and evaluate the implementation of SloMo and was chosen as it is an implementation science framework developed specifically for health care technologies. This paper reports on work focused on reducing complexity in the technology domain. Research to address other NASSS domains relevant to SloMo’s implementation will be reported separately. Intervention complexity has been identified as a key barrier to the implementation of digital technologies in psychosis and, therefore, warrants attention [4]. It is crucial we design digital technology to meet the needs of the end user, particularly in the context of paranoia, where concerns about privacy and technology are often elevated [26].

In summary, the study aimed to optimize the SloMo software for implementation in routine care by addressing limitations of the first release, reducing technology complexity, and improving the user experience for service users and therapists, relative to the first version of the software. We anticipated that through iHCD methods, the likelihood of successful implementation would be enhanced. This will be evaluated further in an ongoing implementation-effectiveness study.

## Methods

### Design

Consistent with the design of the first version of SloMo [16,17] the Double Diamond [8], a human-centered design framework, was used to optimize SloMo for implementation, using inclusive design principles [9]. The Double Diamond is the most widely used co-design framework in the United Kingdom. This framework was selected because of its alignment with inclusive design principles and because it was used to develop the previous release of SloMo [16,17].

### Ethical Considerations

This work was undertaken as a quality improvement project within South London and Maudsley NHS Foundation Trust and, according to institutional policy, did not require review or approval by a research ethics board. Participants provided verbal consent to participate in user-testing sessions and focus groups. Participation was voluntary, and all data were collected anonymously. No identifiable personal or health information was recorded, stored, or analyzed. The project was carried out in compliance with relevant ethical standards for quality improvement initiatives. Participants received gift vouchers for their contributions in line with National Institute for Health and Care Research payment rates [27].

### Software

SloMo (release 1; R1) was self-certified as a class I medical device. We anticipate SloMo release 2 (R2) to be a Class I medical device under Rule 12 (All other active devices; MEDDEV Guideline 2.4/1 Rev 9), as the criteria for Rules 9, 10, and 11 for active devices are not met. The software was programmed by Bitam Ltd, which met ISO (International Organization for Standardization) 13485 and ISO14971 standards (ISO13485 is an international standard for medical device quality management systems; ISO14971 is an international standard for risk management of medical devices). The SloMo therapy platform architecture is compliant with ISO/IEC 27001:2022, 27001:201, ISO/IEC 27017:2015 and 27018:201.

### Sample

To address the risk inherent in participatory design that the most willing, able, and vocal users are more likely to be involved, neglecting the needs of underserved groups, we purposively sampled people from a wide range of backgrounds (ie, across diverse gender, age, ethnicity, technology confidence, and attitudes to therapy). The group was representative of service users across the 3 NHS Trusts, with respect to ethnicity and age (Table 1). Confidence in using technology was self-reported by service user consultants at the start of testing sessions on a scale of “0” (not confident at all) to “100” (very confident). A wide range of technology confidence was self-reported, skewed towards people with higher levels. This is in line with recent literature indicating digital literacy is improving amongst people with psychosis [28].

Therapist patient and public involvement (PPI) consultants currently working with people with psychosis were purposively sampled in relation to gender, age, ethnicity, and experience of delivering psychological interventions for psychosis. Ten therapist PPI consultants participated in the deliver phase. The mean age was 34.9 years (range 26-54) and comprised 7 women and 3 men. One therapist identified as Bengali, 2 as Black British-African, 2 as Black British-Caribbean, 4 as White British, and 1 as White (other). Seven therapists were clinical psychologists or counseling psychologists, one was a high-intensity CBT therapist, two were trainee clinical psychologists, and one was an assistant psychologist. The mean technology confidence score was 86.2 out of 100 (range 70 to 100).

**Table 1:** Demographic characteristics of service user consultants for the develop and deliver phases (N=32).

Characteristics			Value
**Gender, n (%)**			
	Man	18 (56.3)	
	Woman	13 (40.6)	
	Nonbinary	1 (3.1)	
**Age (years), n (%)**			
	18-25	1 (3.1)	
	26-35	12 (37.5)	
	36-45	5 (15.6)	
	46-60	11 (34.4)	
	60+	3 (9.4)	
**Ethnicity, n (%)**			
	Asian or Asian British	3 (9.4)	
	Black British, Caribbean or African	10 (31.3)	
	Mixed or multiple ethnic groups	3 (9.4)	
	White British	15 (46.9)	
	Any other White background	1 (3.1)	
Technology confidence (0-100), mean (range)			82 (10-100)

### Procedure

Transdisciplinary co-design workshops (referred to as “workshops” here forward) were central to the redesign of the new version of SloMo. Our project team included experts by experience (EBEs), designers, clinicians, academics, software developers, video producers, and illustrators (Multimedia Appendix 1 contains further details of the co-design team). Workshops were initially held in person (to support group cohesion), then switched to remote (to facilitate engagement of people from geographically diverse locations). Attendees were informed of the workshop’s focus in advance of the meeting, which was prespecified according to the phase of development. Sessions were recorded so that the discussion could be reviewed, and members who were unable to attend could review and offer feedback via email. A PPI management team oversaw the project.

**Figure 1:**
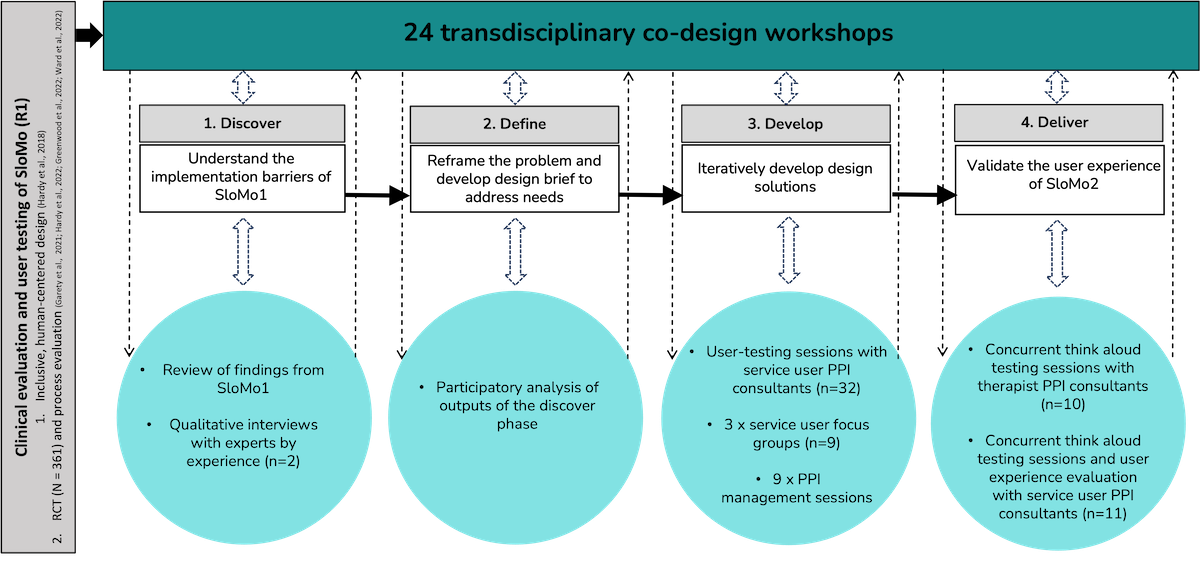
Optimizing SloMo for implementation—design research methods used across the 4 phases of the Double Diamond framework. PPI: patient and public involvement.

The Double Diamond is organized into 4 convergent and divergent phases: discover (understand the problem; divergent), define (reframe the problem and develop a design brief; convergent), develop (develop potential solutions; divergent), and deliver (test out solutions at a small scale and iterate; convergent). It should be noted that divergent phases were necessarily constrained by the previous version of SloMo and its clinical evaluation, and so we adopted a “waterfall-agile” approach [16]. Figure 1 provides an overview of the Double Diamond phases used to optimize SloMo for implementation, which are explained in more detail below.

### Discover

The aim of the discover phase was to develop an understanding of possible implementation barriers for the SloMo software. This first involved reviewing the outputs of the SloMo RCT [15,18-20] in the transdisciplinary co-design workshops, to explore key technology problem areas that could potentially be targeted for the next release of SloMo. In addition, the designers conducted qualitative interviews with 2 EBEs who had received the first release of SloMo. Interviews focused on exploring what was required from a psychological therapy for psychosis, building on insights from Hardy et al [17], as well as exploring how SloMo could be improved.

### Define

The define phase synthesized the insights gathered in the transdisciplinary workshops. The EBEs who provided feedback during the discover phase also took part in the next round of workshops to assist in synthesis. The information from the discover phase was sent to co-design group members to review ahead of time to allow for the opportunity to familiarize and reflect on the materials. Workshops involved reviewing the insights as a group, noting patterns, and sorting into themes, using a group consensus approach. Multiple workshops were run until a clear and workable design brief was developed, in line with identified user needs.

### Develop

The develop phase aimed to generate potential design solutions that helped to address the redefined user needs outlined in the preceding phase. Guided by clinician and EBE recommendations, the designers initially explored content relevant to SloMo (ie, existing apps available on the market, including for mental health, as well as apps in parallel markets, such as meditation, nutrition, and habit-building). The designers fed back their design solutions in co-design workshops, and group consensus determined which ideas were taken forward for iterative development.

Low-fidelity digital prototypes of initial design concepts were developed (Multimedia Appendix 2). Preferred concepts, selected through workshops, were taken for one-to-one user testing with service user PPI consultants. Data from the user testing sessions informed the iterative development of design solutions, leading to the development of higher fidelity digital prototypes of adopted concepts. Prototypes were further iteratively tested and developed through one-to-one service user testing, focus groups with service user and clinician consultants, and workshops. Once a prototype had been validated, it was added to the optimized SloMo minimum viable product (MVP) software, which was taken forward to the deliver phase.

### Deliver

The convergent deliver phase aimed to validate the user experience of the optimized version of SloMo. Given the redesign of SloMo focused on 2 software components (a web-based therapy platform and native mobile app), the deliver phase aimed to validate both through consultation with the applicable user groups. For the web-based therapy platform, therapist PPI consultants were chosen as this would be used by therapists to augment the face-to-face therapy sessions they deliver. Service user PPI consultants were chosen for mobile app feedback, as they were the main intended users of the app.

Concurrent think aloud testing sessions were carried out with therapist PPI consultants. This is a form of usability testing where participants are asked to verbalize their thoughts in real time as they interact with a user interface (UI; ie, modules on the SloMo web-based therapy platform) [29]. Following this, additional unstructured qualitative feedback was gathered. A similar approach was taken for the mobile app. Service user PPI consultants who were involved in the develop phase took part in think-aloud testing sessions. Service users were asked to complete different core functionalities on the app (eg, adding a new worry, slowing down a worry, viewing session summary cards) whilst thinking aloud. Interactions were observed, and qualitative feedback was elicited. Following this, a user experience survey (UES) [19] was completed. This is a 10-item questionnaire (adapted from Lobban et al [30]) with subscales relating to enjoyment, usability, and usefulness. Each item is rated from 0 to 10 and summed for the subscale total scores, which were calculated as percentages, with higher percentages indicating a more positive experience. Following user experience validation, an MVP of SloMo, optimized for implementation, was finalized for release to Apple and Android app stores for piloting clinical use.

## Results

### SloMo Redesign Outputs

The key insights and outputs from each phase of the Double Diamond are presented in Table 2 and expanded on below. Additional details on participants, methods, and outputs can be found in Multimedia Appendix 3 [15,17-20].

**Table 2:** Summary of SloMo redesign using the double diamond framework.

SloMo (R1) RCT^a^ original design solution	Discover: implementation barriers to design solution from SloMo (R1)	Define: design solutions for SloMo (R2) implementation	Develop and deliver: SloMo design outputs
Usable	Poorer usability in men compared with women [15]	UI^b^ that is sufficiently visually appealing	Minimalist redesign of UI Character aesthetics made more realistic
Usable	Lower adherence with less tech confidence [15]	App navigation should feel intuitive	App home screen limited to key functions Embedded onboarding guidance
Usable	Participants disliked carrying 2 phones [20]	Convenient access to mobile app	App availability through Android and iOS app stored
Usable	Therapists struggled pacing sessions^c^	Feedback available on session progress	Session progress bar Burger menu to support navigation
Usable	Difficulty generating goals and safer thoughts^c^	To feel supported in generating content	Co-produced “community” goals and safer thoughts
Trustworthy	Lack of clarity on data transfer^c^	Greater agency over data sharing	Notifications to accept/reject sharing content Ability to select specific content to share/not share “Lock thoughts” function to keep entries private
Enjoyable	Worries on home screen increased distress^c^	To feel supported when opening app	Home screen displays safer thoughts as default Active navigation required to view worries
Personalized	In session progress too slow for some [15,20]	Therapist to tailor sessions to person	Burger menu to tailor navigation Optional in session and between-session tasks
Personalized	Lack of diverse content in vignettes [20]	Manage more paranoia related worries	Co-design of novel vignettes
Normalizing	Negative connotations of “slow” in SlowMo^c^	For SloMo to feel like a positive experience	Therapy name updated to “SloMo”
Normalizing	Lack of intersectional diversity in videos^c^	To feel represented and normalized	Videos conveying a range of protected characteristics Customizable avatar representing the SloMo user
Memorable	Users wanted memory aides post-therapy^c^	SloMo journey to continue after completion	Function to print a “therapy blueprint” Catch-up screen at start of each session Increased repetition of key learning messages

^a^RCT: randomized controlled trial.

^b^UI: user interface.

^c^Barrier identified by patient and public involvement (PPI) consultants in transdisciplinary co-design workshops.

### Discover

Key implementation barriers in the technology domain for the first software version were identified through review of the SloMo RCT outputs and interviews with EBEs. This included reporting of poorer software usability by men and people with lower levels of technology confidence. In addition, qualitative data revealed service users disliked carrying 2 mobile handsets (the first release of SloMo was only available on a study handset). Therapists reported difficulties in pacing the sessions, as there were no signifiers indicating how much session content remained, which aligned with service user feedback indicating progress was at times too slow. Therapists and service users reported finding the content of psychoeducational vignettes limited and instead used SloMo for a greater range of paranoia-related worries than was covered in the software. Other barriers included a lack of clarity around when data was being transferred between devices (ie, the web-based therapy platform and mobile app), an overemphasis on worries instead of safety on the app home screen, difficulties in generating personalized goals and safer thoughts, a lack of diversity across psychoeducational vignettes, and forgetting content covered in therapy sessions once these had ended. Outputs at the end of the discover phase included a list of technology-related implementation barriers. These barriers were reviewed during transdisciplinary workshops and taken forward to the define phase.

### Define

The define phase reaffirmed the importance of SloMo’s design brief of users needing a form of CBTp that was usable, trustworthy, enjoyable, personalized, normalizing, and memorable [17]. The problems identified during the discover phase were mapped onto these domains, indicating these were areas that could be targeted in the next iteration to further deliver on this design brief and enhance user experience.

The issues outlined in the discover phase were reframed as user needs through co-design workshops. Service users needed a visually appealing UI, an intuitive mobile app navigation, and convenient access to support usability. To foster a sense of trust, service users required greater agency over data sharing to and from the web-based therapy platform and mobile app. Service users needed to feel psychologically supported when opening the mobile app, for SloMo to feel like a positive experience, and to feel represented throughout. There was a need for learning ways of managing a wider range of paranoia-related worries, and for the SloMo journey to continue once face-to-face sessions had ceased. For therapists, it was important that they were provided feedback on session progress so they could pace sessions efficiently. There was a need for therapists to be able to use the web-based therapy platform flexibly, allowing them to tailor sessions to the client. The outputs at the end of the define phase included a design brief of the solution, which specified the user needs to be addressed in relation to the original design definition, which informed the subsequent development phase.

### Develop

Thirty-two 1:1 service user testing sessions and 3 service user focus groups (n=9) were carried out. Initial user testing focused on design concepts, and later stages focused on interactive digital prototypes. Initial design concepts taken to 1:1 user testing sessions included (1) aesthetics of bubbles used to convey worry and safer thoughts, (2) design of the avatar representing the SloMo user on their journey, (3) SloMo character illustrations, and (4) aesthetics of physical tip cards used as a memory aid. Other concepts (eg, lived experience vignettes, UI changes, data transfer updates) were iteratively developed in co-design workshops. Multimedia Appendix 4 displays the updated concepts for the new version of SloMo compared with the previous one used in the RCT.

**Figure 2:**
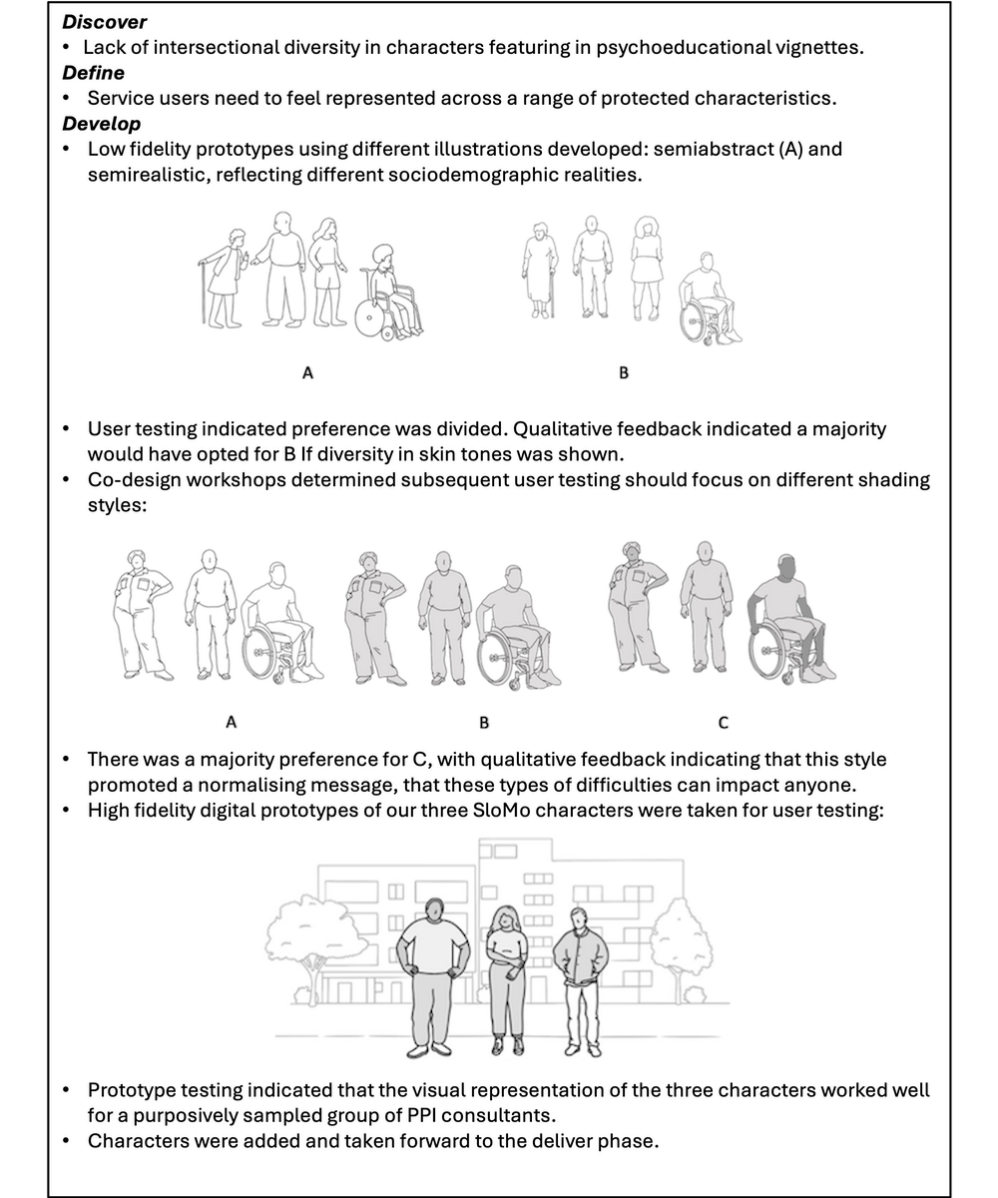
Development of SloMo characters featuring in psychoeducational vignettes. PPI: patient and public involvement.

**Figure 3:**
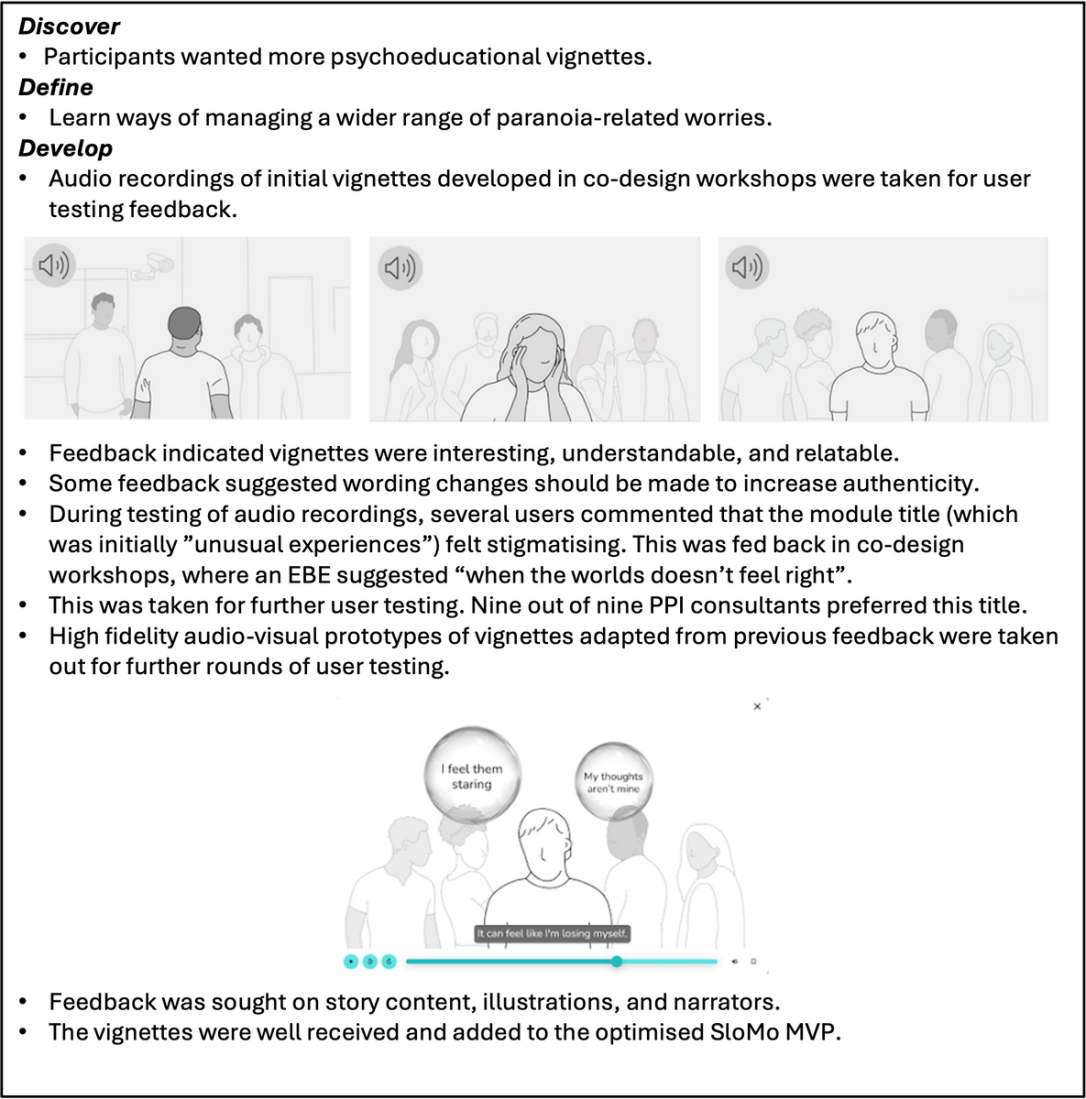
Co-designing psychoeducational vignettes on anomalous experiences and paranoia. EBE: experts by experience; MVP: minimum viable product; PPI: patient and public involvement.

**Figure 4:**
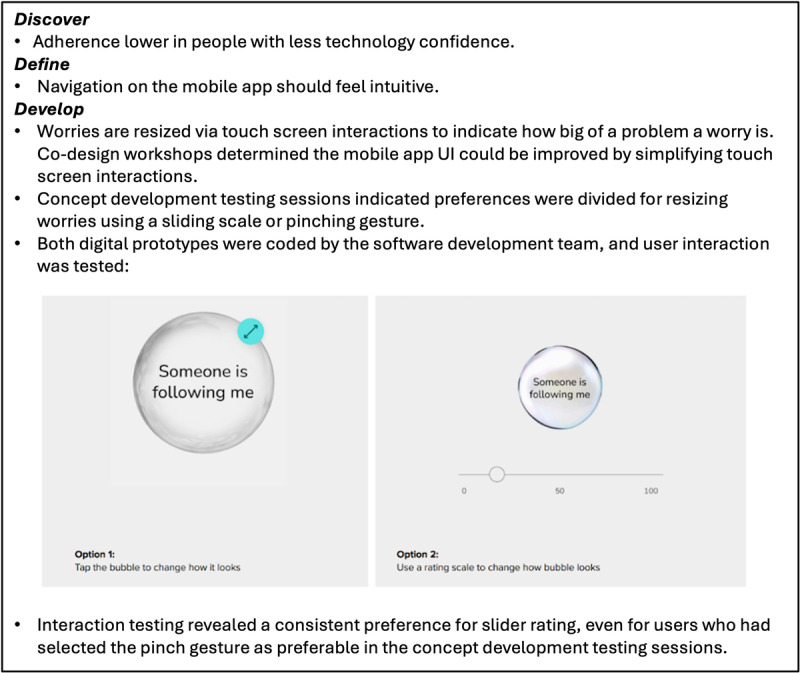
Bubble interactions on the mobile app to indicate levels of distress.

High-fidelity interactive digital prototypes developed and taken out for individual user testing included (1) user interactions with thought bubbles, (2) user interactions for building a customizable avatar, (3) character illustrations featured in audio-visual psychoeducational vignettes, and (4) the content of newly developed psychoeducational vignettes. User testing feedback in both the concept and prototype development phases was discussed in co-design workshops and iteratively developed until there was a group consensus that the prototype should be adopted. Figures 2 to 4 provide an overview of the development of specific SloMo features from the discover to deliver phase. Multimedia Appendix 5 provides additional data collected during the develop phase.

Outputs at the end of the develop phase included iteratively developed digital design solutions addressing the user needs and design brief from the define phase. In addition, early-stage demonstration and interaction testing of design solutions with user testing consultants provided preliminary validation of specific design solutions. These were then developed into the SloMo (R2) MVP during the deliver phase.

### Deliver

Table 3 presents quotes from the therapist PPI consultants during the think-aloud testing sessions. Overall, the web-based therapy platform received positive feedback. Therapists appreciated the platform’s simplicity and visual appeal, believing it would enhance the enjoyment and engagement of sessions. They noted therapeutic benefits, such as providing more structure to sessions and making therapy feel more personalized. Additionally, therapists found the platform engaging and flexible, allowing for adaptation based on various therapeutic needs. The addition of progress bars was positively commented on as a helpful feature for therapists to manage the session time more effectively.

During the think-aloud sessions, several areas for improvement were identified. Some therapists expressed hesitation about navigating back and forth due to concerns that personalized content entered into the platform might be lost. Others suggested that enjoyment could be further enhanced by incorporating more gamification elements, such as badges or achievements, to boost engagement. Additionally, there were recommendations to increase inclusivity by adding different languages, enabling access for users whose first language is not English.

Eleven service user PPI consultants who had previously taken part in user testing feedback sessions in the development develop phase took part in the concurrent think-aloud testing sessions for the deliver phase. Think-aloud consultants were purposively sampled to be representative of the psychosis population across the respective areas of the 3 trusts. The sample included 7 women and 4 men, whose ages ranged from 25 to 60 years, and 4 consultants identified as Black British, 1 as South American, and the remaining 6 as White British.

The UES findings for each subscale are presented in Table 4. The ratings suggest a good user experience of the SloMo demonstration, with a total UES mean of 83.4%. Notably, the mean score for enjoyment was around 10% lower than the domains of usability and usefulness, as well as showing a greater range of scores. This suggested that improved enjoyment may be an area requiring further development.

**Figure 5:**
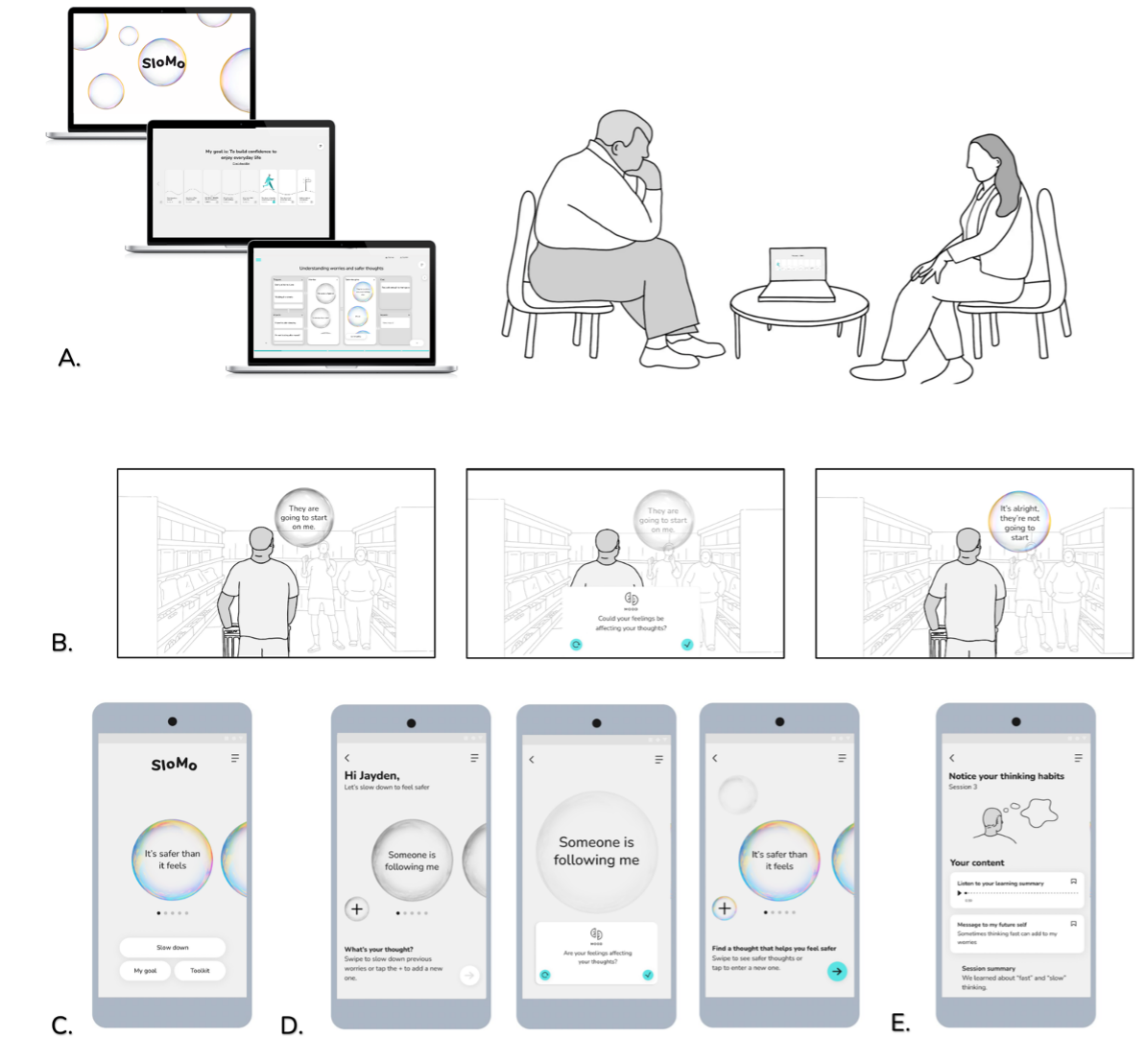
SloMo sessions with a therapist (in person or online) are supported by a (A) web-based therapy platform which promotes (B) understanding and managing worries through lived experience stories and interactive tasks. Personalized thought bubbles are visualized and synchronized with the (C) SloMo mobile app, (D) then “slowed down” to find ways of feeling safer. Additional mobile app features can be accessed via a toolkit, which includes (E) personalized learning summaries of therapy sessions (reproduced from Hardy et al [16], which is published under Creative Commons Attribution 4.0 International License [31]).

Table 5 displays qualitative feedback captured during think-aloud user testing sessions with service user PPI consultants. The feedback aligned with the UES ratings, indicating areas across the 3 domains of user experience that were well received. Interestingly, there were suggestions around how enjoyment could be enhanced, possibly providing insight into lower UES scores for this domain. For instance, haptic feedback was seen as a feature that could boost enjoyment, and improving touch screen interactions could further enhance usability. Outputs at the end of the develop phase included an MVP of SloMo (R2) that had received early-stage user experience validation from service users and therapist consultants. The user experience of the software will be further evaluated in an ongoing implementation-effectiveness study. Figure 5 [16,31] illustrates the prototype delivered from the develop phase.

**Table 3:** Quotes from the therapist consultants (n=10) during think-aloud testing.

Theme	Quote
Enjoyment	I like the visuals… It is attractive to use The gamified elements make it more enjoyable This is definitely more enjoyable and accessible than conventional CBT for psychosis
Usability	I like how simple it is… It’s a nice scaffolding tool I like the bar along the bottom to help with time management I feel more confident to be flexible given my experience level... I worry that low intensity therapists would struggle to deliver in a flexible manner
Usefulness	The structure scaffolds good CBT… you can be more focused on the person in the room The formulation feels personalized It gives such a clear focus for both therapists and client I think it makes [the therapy process] less intense for people who struggle with conversations for an hour

**Table 4:** User experience survey (UES) ratings of SloMo from service user consultants (n=11).

UES variable		Values
**Enjoyment**		
	Mean % (SD)	76.1 (17.3)
	Range	47.5-100.0
**Usability**		
	Mean % (SD)	85.8 (8.5)
	Range	72.5-100.0
**Usefulness**		
	Mean % (SD)	87.0 (8.5)
	Range	67.5-100.0
**Total UES**		
	Mean % (SD)	83.4 (9.7)
	Range	69.2-95.8

**Table 5:** Quotes from service user consultants (n=11) during think aloud testing sessions.

Theme	Quote
Enjoyment	I really liked the safer thoughts and the colours on the positive safer thoughts Using haptics would be good… adds another layer The swiping [screen interaction] is difficult in places. I would find this frustrating
Usability	Ease of access is the biggest positive It's not difficult to add worries and sometimes people are afraid of technology, but I think it’s not difficult to use this app I feel like I can navigate around it and haven’t spent much time on in… It's pretty intuitive
Usefulness/Acceptability	I like you don’t need to share all of your thoughts with the therapist …sometimes you don’t want to show your thoughts to other people I like that it’s really personalised, I can’t count the amount of times that I have been given something that has not been in my own words in therapy

## Discussion

### Principal Results

This study optimized the SloMo therapy software for implementation in routine NHS care, through reducing technology complexity and boosting user experience [24,25]. An iHCD was used, involving transdisciplinary co-design workshops, focus groups, one-to-one user testing, and think-aloud testing sessions. From this research, we have produced an optimized MVP of SloMo, which is now being tested in the SloMo2 hybrid implementation-effectiveness study (ClinicalTrials.gov identifier: NCT06568081). User testing indicated that users need a form of CBTp that is usable, trustworthy, enjoyable, personalized, normalizing, and memorable, in line with earlier work from our team [17]. Design targets for improving the SloMo software were identified to deliver this design brief and address user needs. For reducing complexity, key targets included simplified app interfaces to highlight core functionalities, improved navigation, and, for boosting user experience, this included providing users with greater agency over data, and improving the representation of protected characteristics, including their intersectionality. The user experience of this optimized version of SloMo may be improved relative to the first version that was tested in the SloMo RCT, indicated by higher UES scores compared with those reported by Hardy et al [19].

A distinctive aspect of our product development approach is the integration of lived experience involvement in all stages of the design work. Traditional approaches to intervention development in clinical research often follow a convergent design process using existing theory and evidence to determine intervention content and structure. This approach can miss novel opportunities to improve the user experience of therapies. In contrast, we adopted a “waterfall-agile” approach to facilitate the co-design of SloMo as an evidence-based digital therapeutic [16]. This allowed for agility in iterating specific features (eg, UI, psychoeducational vignettes) while maintaining fidelity to the previous UI and therapy protocol [18]. To support this, we embraced divergence, revisiting and challenging earlier design assumptions in line with iHCD principles. An example of this was the service user feedback that “unusual experiences” could be perceived as stigmatizing, despite this wording being widely used in CBTp [32] and the identification of a term describing these types of experiences, which felt more relevant and acceptable—“when the world doesn’t feel right.” Despite this divergent approach, we were necessarily constrained in certain respects. The need to build on a preexisting evidence base meant that some design decisions were necessarily limited by prior research findings and the parameters of the therapeutic model. For instance, some users suggested moving away from visualizing thoughts and thinking habits as bubbles, which was not possible, as this is a core component of SloMo and its previous clinical evaluation.

### Limitations

Resource constraints restricted our ability to optimally develop this new version of SloMo. This included time pressure, as there was a need to produce an MVP in line with our implementation-effectiveness study milestones. Another constraint was workforce access, such as access to PPI consultants (therapists and service users), designers, and software developers. These limitations meant that pragmatic decisions had to be made for the redesign, for instance, regarding the types of software changes that could be made and what features were selected for user testing. Whilst decisions were made collaboratively within the transdisciplinary co-design workshops, it is possible that different design decisions may have been made with additional resources. These constraints may be viewed as a barrier to reducing complexity in the technology domain of the NASSS framework [24,25]. Ideally, future projects could be based in “living labs,” whereby lived experience, clinical, technological, industry, regulatory, and research expertise is brought together in an organizational structure, with coproduction underpinning the co-design of digital health [33].

A limitation of the PPI consultant validation is that the service user and therapist experience of the optimized version of SloMo was based on a relatively brief exposure to the software, and so may not be directly comparable to the user experience findings from the SloMo RCT. Further, the PPI consultants involved in the delivery phase had been involved in the SloMo design work for an extended period and may have been less independent than the RCT sample. This may limit the conclusions we can draw from this early-stage validation. However, more rigorous data will be provided in our ongoing implementation study, examining SloMo when delivered by frontline therapists.

Validation of the SloMo (R2) MVP with service user consultants found enjoyment was rated lower than other domains on the UES [17], indicating an aspect of user experience that warrants attention in future development. Qualitative feedback highlighted specific areas for improvement, such as incorporating haptic feedback and improving user interaction. The digital therapy engagement literature could inform additional design strategies [34]. Nonetheless, lower enjoyment scores may reflect comparisons with nontherapeutic commercial apps, where higher levels of enjoyment are likely expected. It will be important to monitor this throughout the implementation-effectiveness study, and iterate on the software as needed; for example, monitoring engagement may act as a proxy for enjoyment.

### Conclusions and Future Directions

This study demonstrates the value of using iHCD to optimize DMHIs for implementation in routine mental health care. By adopting an iterative, transdisciplinary approach that prioritized collaboration with service users, therapists, and technical experts, the SloMo redesign addressed critical usability barriers, fostered inclusivity, and enhanced the therapy’s ability to meet the diverse needs of its target population. This methodology offers a valuable model for future mental health innovation, where the needs of diverse user groups are addressed, and therapeutic technologies are designed to be impactful and sustainable in routine care settings. We are now evaluating the implementation and effectiveness of the optimized version of SloMo when delivered by routine care therapists. We envisage SloMo will continue to be iterated based on learning from this work, in line with the principles of implementation science [35].

## Appendix

Multimedia Appendix 1 Codesign team involved across Double Diamond phases.

Multimedia Appendix 2 Digital prototypes used in user testing sessions during develop phase.

Multimedia Appendix 3 Participants, methods, and outputs for subphases of the Double Diamond redesign process.

Multimedia Appendix 4 Comparison of design outputs from the develop phase, compared to SloMo (release 1).

Multimedia Appendix 5 Examples of qualitative feedback from service user PPI consultants during the develop phase.

## Abbreviations

CBTp: cognitive behavioral therapy for psychosis
EBE: experts by experience
iHCD: inclusive, human-centered design
ISO: International Organization for Standardization
MVP: minimum viable product
NASSS: Non-adoption, Abandonment, Scale-up, Spread, Sustainability
NHS: National Health Service
PPI: patient and public involvement
RCT: randomized controlled trial
UES: user experience survey
UI: user interface
