# Supplementary material for: Optimizing SloMo, a Digitally Supported Therapy Targeting Paranoia, for Implementation: Inclusive, Human-Centered Design Study
Source: JMIR Hum Factors. 2025 Dec 22;12:e75377. doi: 10.2196/75377 (PMC12770921; doi:10.2196/75377)
Supplement: Multimedia Appendix 1 [file humanfactors_v12i1e75377_app1.docx]

|  | | | | | |
| --- | --- | --- | --- | --- | --- |
| **Role** | **Discover** | **Define** | **Develop** | **Deliver** | |
| ***Manufacturer (****Kings College London)* |  | | | | |
| Project Lead / Co-founder / Clinical Academic Psychologist | 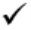 | 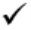 | 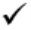 | 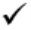 | |
| Co-founder / Clinical Academic Psychologist | 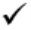 | 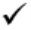 | 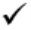 | 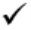 | |
| Co-founder / Clinical Academic Psychologist | 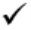 | 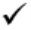 | 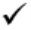 | 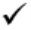 | |
| Product Owner / Clinical Academic Psychologist | 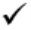 | 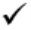 | 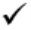 | 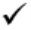 | |
| Assistant Psychologist | 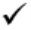 | 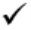 | 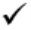 | 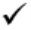 | |
| Regulatory, Clinical Safety & Compliance Consultant | 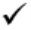 | 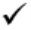 | 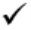 | | 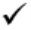 |
| ***Lived experience*** *(SLaM, SPFT & CNTW NHS Trusts)* | | | | | |
| Lead PPI Consultant | 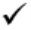 | 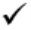 | 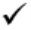 | | 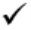 |
| Lead PPI Consultant | 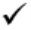 | 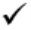 | 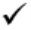 | | 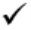 |
| Expert by experience | 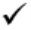 | 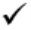 | 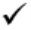 | | 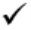 |
| Expert by experience | 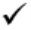 | 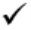 | 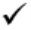 | | 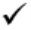 |
| Service user consultants (n=32) |  |  | 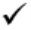 | | 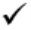 |
| Therapist consultants (n=10) |  |  |  | | 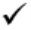 |
| ***Software Design*** *(Special Projects)* |  | | | | |
| Design Contract Manager | 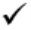 | 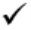 | 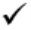 | 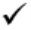 | |
| Creative Director | 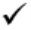 | 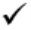 | 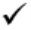 | 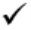 | |
| Design Lead | 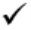 | 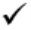 | 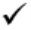 | 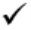 | |
| Designer | 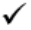 | 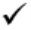 | 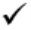 | 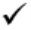 | |
| ***Software Development*** *(Bitjam Ltd)* |  | |  |  | |
| Development Contract Manger (Managing Director) |  | 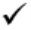 | 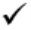 | 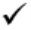 | |
| Technical Lead (Software Developer) |  | 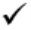 | 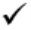 | 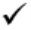 | |
| Software Developer |  | 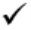 | 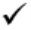 | 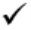 | |
| Software Developer |  | 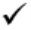 | 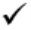 | 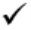 | |
| ***Video Production*** *(Carse & Waterman)* |  | | | | |
| Production Contract Manager |  |  | 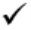 |  | |
| Production Assistant |  |  | 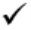 |  | |
| Animator |  |  | 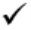 |  | |
| ***Illustration Production*** |  | | | | |
| Illustrator |  | 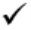 | 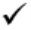 | |  |
|  | | | | | |
