# Supplementary material for: Optimizing SloMo, a Digitally Supported Therapy Targeting Paranoia, for Implementation: Inclusive, Human-Centered Design Study
Source: JMIR Hum Factors. 2025 Dec 22;12:e75377. doi: 10.2196/75377 (PMC12770921; doi:10.2196/75377)
Supplement: Multimedia Appendix 2 [file humanfactors_v12i1e75377_app2.docx]

| **Feature** | **Example testing material** |
| --- | --- |
| Bubble interaction | 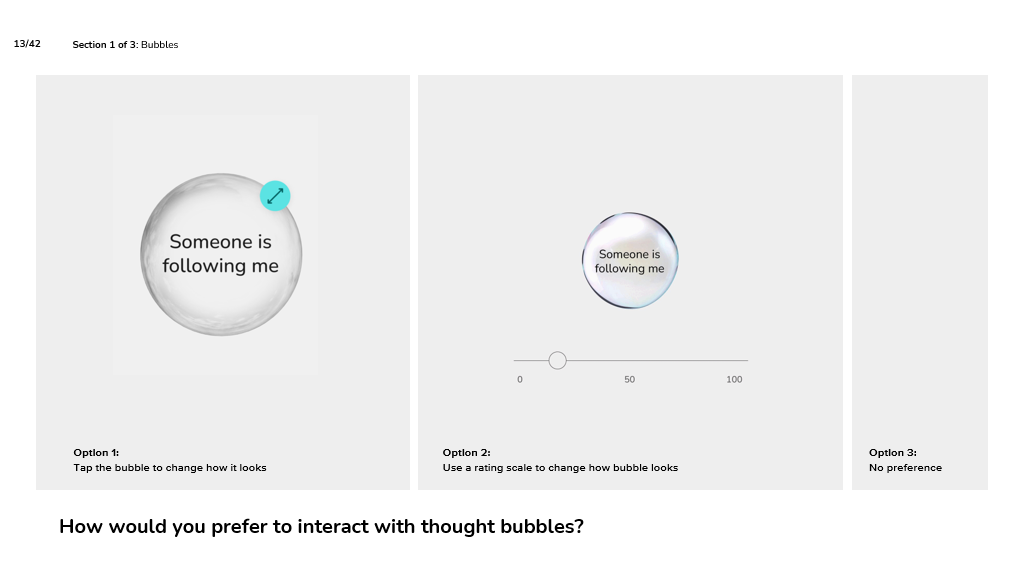 |
| SloMo characters | 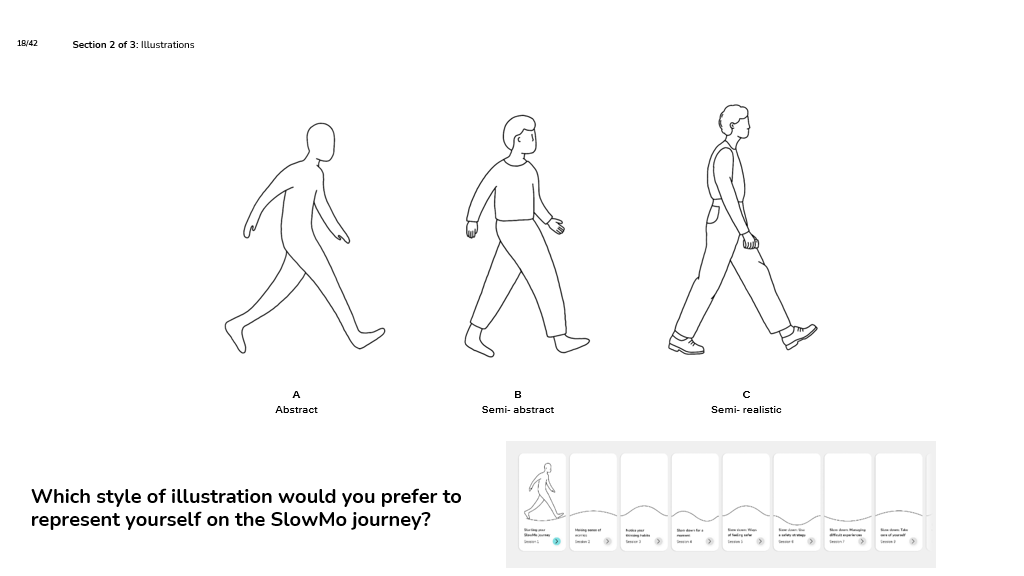 |
| SloMo avatar | 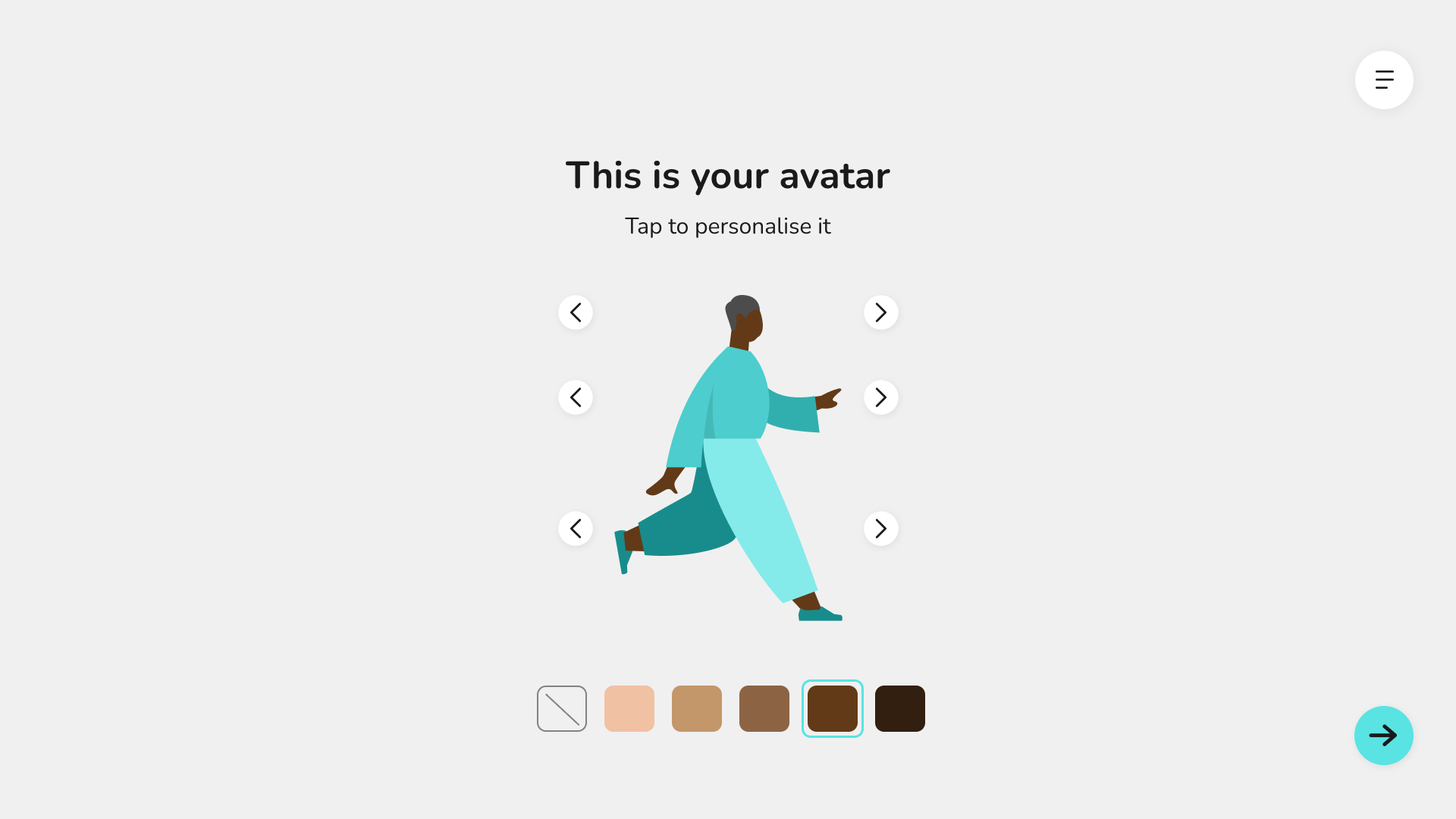 |
| Novel stories | 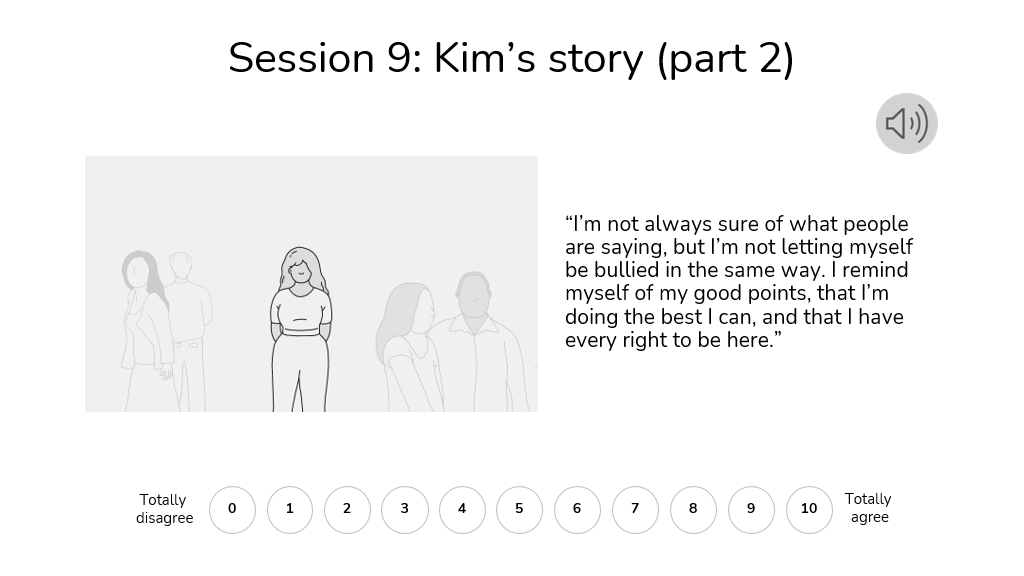 |
|  |  |
