# Supplementary material for: Optimizing SloMo, a Digitally Supported Therapy Targeting Paranoia, for Implementation: Inclusive, Human-Centered Design Study
Source: JMIR Hum Factors. 2025 Dec 22;12:e75377. doi: 10.2196/75377 (PMC12770921; doi:10.2196/75377)
Supplement: Multimedia Appendix 3 [file humanfactors_v12i1e75377_app3.docx]

| **Phase** | **Participants** | **Methods** | **Outputs** |
| --- | --- | --- | --- |
| **Discover** | Clinical, academic, design & software team. | **Desk research of SloMo (R1) findings** – RCT ([1]; n=361), therapy delivery ([2]; n=140), user experience ([3];n=168) and therapy experience ([4]; n=22) | - Identified implementation barriers to meeting the original definition of SloMo’s design solution. |
|  | Experts by experience (EBEs) (n=2) | **Qualitative interviews** –design team conducted open-ended interviews with EBEs who had received SloMo (R1). |  |
| **Define** | Codesign team (clinical, academic, lived experience, and software). | **Participatory analysis** –technology related implementation barriers identified in Discover phase were reviewed and thematically synthesised. | - Implementation barriers redefined into user needs. - Design solution defined as: usable, trustworthy, enjoyable, personalised, normalising, and memorable. |
| **Develop** | Service user consultants (n=32) | **User testing sessions –**design solutions tested and mixed methods data collected. | - Redesigned functions and content - Validation of design outputs. - Early-stage validation of design solutions. - Design solutions added to SloMo (R2) MVP |
|  |  | **3 x focus groups –** high fidelity prototypes of design solutions used by consultants to offer feedback and assess early-stage usability. |  |
| **Deliver** | Therapist (n=10) &  Service user (n=11) consultants | **Think aloud testing sessions –** Users navigated around MVP of SloMo (R2), thinking aloud as they did so (prompted by manufacturer team member). | - Qualitative insights on user experience. - Feedback indicated software was enjoyable, usable & acceptable. |
|  | Service user consultants (n=11) | **User Experience Survey** [5] **–** 10-item questionnaire with subscales relating to enjoyment, usability and acceptability. | - User experience of SloMo (R2) MVP was improved relative to SloMo (R1). |
|  | | | |

**References**

1. Garety P, Ward T, Emsley R, Greenwood K, Freeman D, Fowler D, Kuipers E, Bebbington P, Dunn G, Hardy A. Digitally supported CBT to reduce paranoia and improve reasoning for people with schizophrenia-spectrum psychosis: the SlowMo RCT. Effic Mech Eval 2021 Aug 12;8(11):1–90. doi: 10.3310/eme08110

2. Ward T, Hardy A, Holm R, Collett N, Rus-Calafell M, Sacadura C, McGourty A, Vella C, East A, Rea M, Harding H, Emsley R, Greenwood K, Freeman D, Fowler D, Kuipers E, Bebbington P, Garety P. SlowMo therapy, a new digital blended therapy for fear of harm from others: An account of therapy personalisation within a targeted intervention. Psychol Psychother Theory Res Pract 2022;95(2):423–446. doi: 10.1111/papt.12377

3. Hardy A, Ward T, Emsley R, Greenwood K, Freeman D, Fowler D, Kuipers E, Bebbington P, Garety P. Bridging the Digital Divide in Psychological Therapies: Observational Study of Engagement With the SlowMo Mobile App for Paranoia in Psychosis. JMIR Hum Factors 2022 July 1;9(3):e29725. doi: 10.2196/29725

4. Greenwood KE, Gurnani M, Ward T, Vogel E, Vella C, McGourty A, Robertson S, Sacadura C, Hardy A, Rus-Calafell M, Collett N, Emsley R, Freeman D, Fowler D, Kuipers E, Bebbington P, Dunn G, Michelson D, Garety P, the SlowMo Patient PI (PPI) team. The service user experience of SlowMo therapy: A co-produced thematic analysis of service users’ subjective experience. Psychol Psychother Theory Res Pract 2022;95(3):680–700. doi: 10.1111/papt.12393

5. Hardy A, Wojdecka A, West J, Matthews E, Golby C, Ward T, Lopez ND, Freeman D, Waller H, Kuipers E, Bebbington P, Fowler D, Emsley R, Dunn G, Garety P. How Inclusive, User-Centered Design Research Can Improve Psychological Therapies for Psychosis: Development of SlowMo. JMIR Ment Health 2018 Dec 5;5(4):e11222. doi: 10.2196/11222
