# Supplementary figures and images for: Optimizing SloMo, a Digitally Supported Therapy Targeting Paranoia, for Implementation: Inclusive, Human-Centered Design Study
Source: JMIR Hum Factors. 2025 Dec 22;12:e75377. doi: 10.2196/75377 (PMC12770921; doi:10.2196/75377)

|  | **SloMo R1** | **SloMo R2** |
| --- | --- | --- |
| **Worry bubble** | 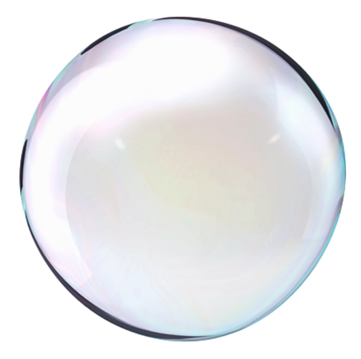 | 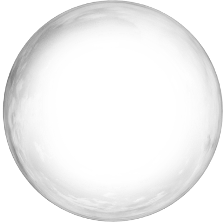 |
| **Safer thought bubble** | 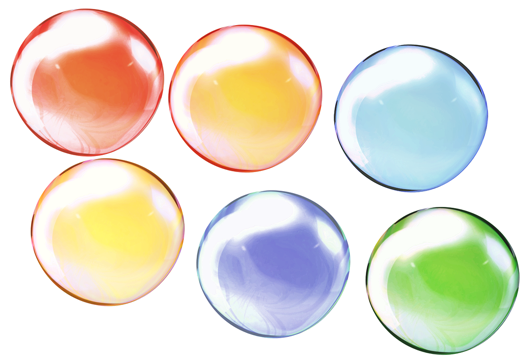 | 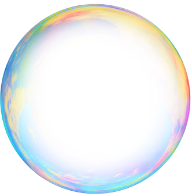 |
| **Avatars** | 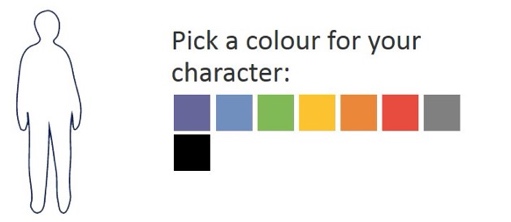 | 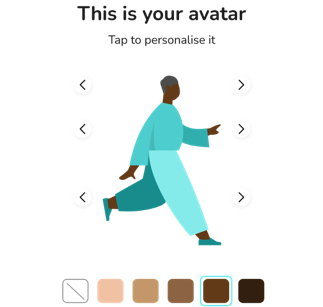 |
| **Characters** | 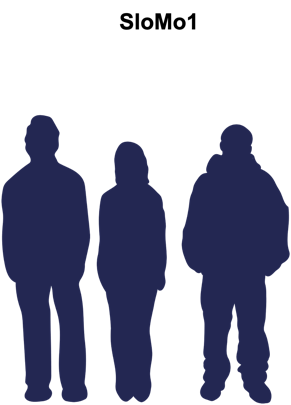 | 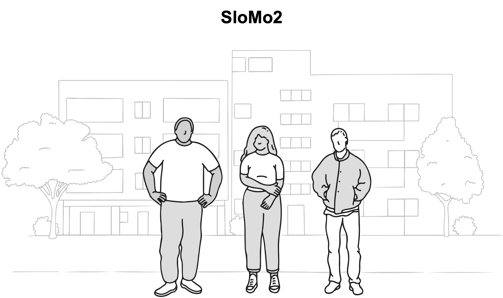 |
| **Tip cards** | 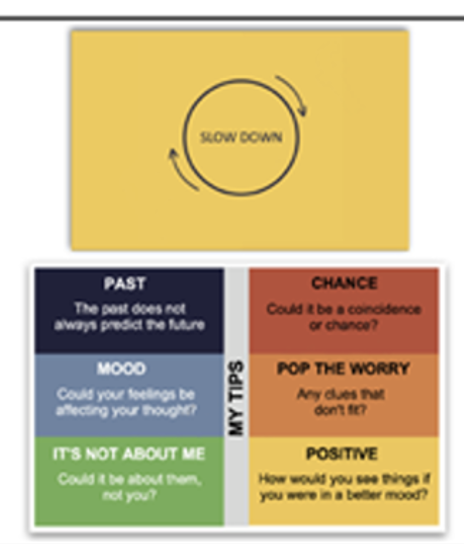 | 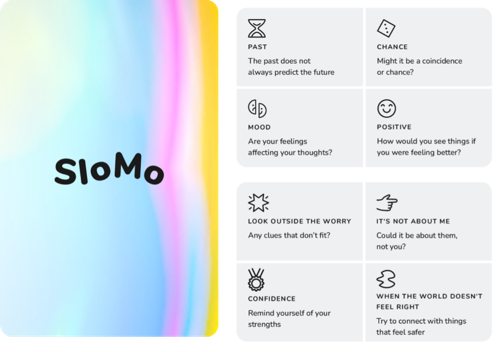 |

Supplement: Multimedia Appendix 4 [file humanfactors_v12i1e75377_app4.docx]
