# Supplementary material for: Optimizing SloMo, a Digitally Supported Therapy Targeting Paranoia, for Implementation: Inclusive, Human-Centered Design Study
Source: JMIR Hum Factors. 2025 Dec 22;12:e75377. doi: 10.2196/75377 (PMC12770921; doi:10.2196/75377)
Supplement: Multimedia Appendix 5 [file humanfactors_v12i1e75377_app5.docx]

| **SloMo feature** | **Examples of user testing feedback** |
| --- | --- |
| **Therapy name** | *“I always had an issue towards ‘Slow’…Reminds me of people getting bullied in playground”* |
|  | *“Negative connotations of slow, being slower, talking slower – not a positive, ‘slo’ is shorter and better”* |
| **Thought bubbles** |  |
| ***Visual concept*** | *“The grey colour feels too light, needs to be darker to reflect worries”* |
|  | *“[The new version of SloMo] feels more appropriate to my darker thoughts”* |
|  | *“The colours bring instant joyful feelings, feels safer”* |
| ***Interaction*** | *“It’s great so long as there will be enough information for the purpose of enlarging it”* |
|  | *“It’s simpler, easier to scale”* |
| **Avatars** |  |
| ***Visual concept*** | *“It’s important for me to be able to see myself in avatar, so it’s close to me and [the] journey”* |
|  | *“Having disability aids helps people feel seen and included”* |
|  |  |
| ***Building*** | *“It feels just right”* |
|  | *“Slick… [it was] easy to use the arrows to select different features”* |
| **SloMo characters** | *“Important not to whitewash…don’t represent [just] white skin tones”* |
|  | *“I identified with [Jayden], being a Black male”* |
|  | *“The regional accents are nice”* |
|  | *“The names sound quite English. Would be nice to have more international sounding names”* |
| **Audio visual stories** | *“I really connect with [this vignette]. Potent and real – things that people wouldn’t always say”* |
|  | *“I don't like that the black man's story is always the just handle it. For men they're always told just be strong, toughen up, you can handle it”* |
|  | *“It’s like reading excerpts from my own life”* |
| **Tip cards** | *“Useful for if people can’t or won’t use tech”* |
|  | *“Like that it’s anonymous”* |
